# Progress in Data Acquisition of Wearable Sensors

**DOI:** 10.3390/bios12100889

**Published:** 2022-10-18

**Authors:** Zixuan Liu, Jingjing Kong, Menglong Qu, Guangxin Zhao, Cheng Zhang

**Affiliations:** College of Engineering, Nanjing Agricultural University, Nanjing 210031, China

**Keywords:** wearable sensors, data acquisition, signal conditioning, analog-to-digital conversion

## Abstract

Wearable sensors have demonstrated wide applications from medical treatment, health monitoring to real-time tracking, human-machine interface, smart home, and motion capture because of the capability of in situ and online monitoring. Data acquisition is extremely important for wearable sensors, including modules of probes, signal conditioning, and analog-to-digital conversion. However, signal conditioning, analog-to-digital conversion, and data transmission have received less attention than probes, especially flexible sensing materials, in research on wearable sensors. Here, as a supplement, this paper systematically reviews the recent progress of characteristics, applications, and optimizations of transistor amplifiers and typical filters in signal conditioning, and mainstream analog-to-digital conversion strategies. Moreover, possible research directions on the data acquisition of wearable sensors are discussed at the end of the paper.

## 1. Introduction

**Figure 1:**
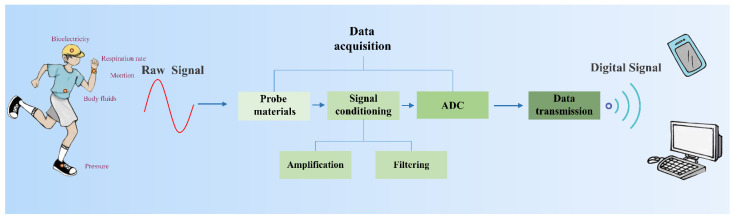
Wearable sensor working processes of wearable sensors.

**Figure 2:**
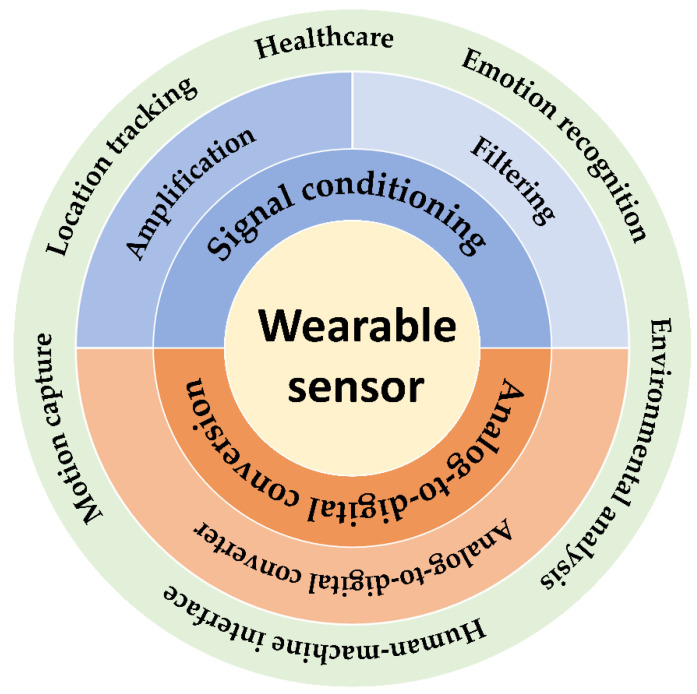
Figure of contents, including amplifiers pursuing high linearity and low power consumption; filters suitable for signal and noise characteristics; high-performance ADCs with both noise rejection and energy efficiency.

Wearable sensors have brought great convenience to health monitoring [1,2,3], medical treatment [4], human-machine interface [5,6], and so on. As shown in Figure 1, wearable sensors are able to monitor various human body signals, ranging from biophysical signals (including human motions, respiration rates, bioelectricity, etc.) to biochemical signals (such as body fluids, blood components, glucose, etc.). The target biophysical/biochemical signals are converted to electrical signals by probes, the first component of the data acquisition (DAQ) module. Then, the raw electrical signals are processed by the remaining two components of the DAQ module, i.e., signal conditioning and analog-to-digital conversion in sequence. The processed signals are eventually transmitted to terminals by the data transmission (DT) module. Thus, signal conditioning and analog-to-digital conversion modules are as indispensable as probes for wearable sensors. Currently, most research on wearable sensors focuses on the development of probe materials with properties of high stretchability [7,8], optical transparency [9], hydrophobicity [10], air permeability [11], biocompatibility [12], conductivity [13,14], and sensitivity [15,16,17,18,19]. There are numerous reviews focusing on sensor materials [20,21,22]; however, less research is systematically focused on signal conditioning and analog-to-digital conversion. This paper highlights the signal conditioning methods and the designs of the analog-to-digital convertor (ADC) in wearable sensors (Figure 2).

Wearable sensors are mainly used to monitor a variety of human body signals which are relatively weak (such as bioelectricity, pulse, body fluid, etc.) and are easily interfered by high-frequency noise, electrode contact noise, power frequency, and so on [23]. Therefore, a powerful signal conditioning unit to amplify the extracted target signals and filter the noise is strongly desired. In this unit, an amplifier enlarges the analog signal collected by the probe and initially suppresses common mode noise. An appropriate filter with a specific passband and stopband is then employed to filter the noise further. After completing these processes, the analog nature of the target signal is extracted. Nevertheless, it cannot be directly recognized by the computer. Thus, an ADC undertakes the important task of converting an analog signal into a recognizable digital signal. During the conversion, ADC is required to minimize the signal loss. After the conversion, the sensor has completed the signal conditioning and analog-to-digital conversion, producing a valuable signal. The signal will be transmitted to a target end, such as a computer and mobile phone, for further processing and analysis.

In the signal conditioning section, the discussion begins with the optimizations of the transistor amplification circuit, including the energy efficient design and common-mode rejection ratio (CMRR) enhancement. The following sections show three filters commonly used in wearable sensors, summarizing their filtering characteristics and applicability, and introduce the latest filter optimization research. In the analog-to-digital conversion section, we highlight the most suitable successive approximation register (SAR) ADCs and the commonly used sigma-delta ADCs for wearable devices, and review the linearization enhancement strategies and energy efficient methods for ADCs. Finally, the discussions are concluded with an overview of challenges encountered in the current wearable sensor designs and possible future directions.

## 2. Signal Conditioning

The signals monitored by sensors worn on the human body are often weak and susceptible to external noise and human motion artifacts. Signal conditioning is the process of amplifying the target signal while filtering noise and artifacts, then classifying and extracting characteristic signals [24] which can be acquired by an ADC.

### 2.1. Amplification

The human body signals collected by wearable sensors are composed of analog signals with the characteristics of low intensity, low frequency, and narrow bandwidth. These irregular, weak, and noisy signals are difficult to be processed by subsequent modules. It requires an amplification circuit to increase the amplitude of the raw signal. Amplification is an important part of wearable sensors which has been systematically studied. These studies strive to minimize the circuit area and power consumption while maximizing the CMRR.

**Figure 3:**
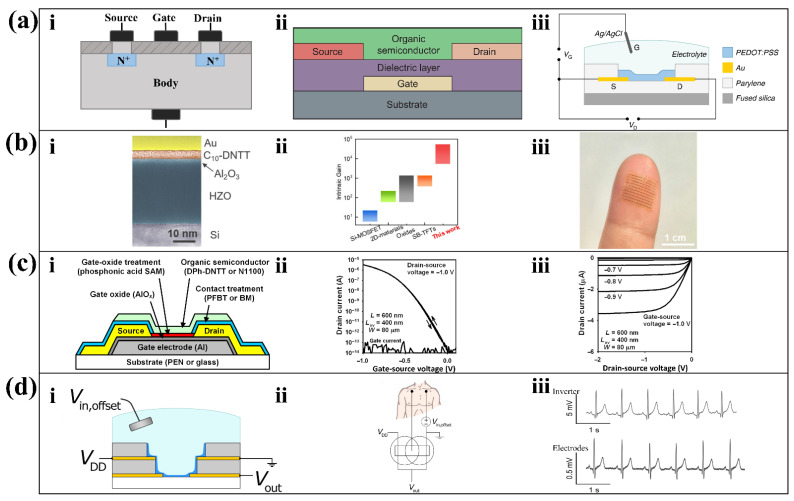
Transistor structures and optimized designs. (**a**) The structures of three transistors. Structure of classical insulated gate type FET (i); OTFT (ii) [34]; reproduced under the terms of the CC-BY 4.0 Creative Commons Attribution License, Copyright 2004, The Authors, published by Elsevier; and OECT (iii) [35]; reproduced under the terms of the CC-BY 4.0 Creative Commons Attribution License, Copyright 2013, The Authors, published by Springer Nature. (**b**) Sub-thermionic organic transistors. (i) False-color cross-section transmission electron microscopy image of the transistor. (ii) Comparison of intrinsic gain among different TFT technologies. (iii) An array of flexible devices that fit on your fingertips [36]. Reproduced under the terms of the CC-BY 4.0 Creative Commons Attribution License, Copyright 2021, The Authors, published by Springer Nature. (**c**) Nanoscale flexible OECT (i) with high on/off current ratio up to 3 × 10^8^ (ii) and sub-threshold swing down to 70 mV/decade (iii) [37]. Reproduced under the terms of the CC-BY 4.0 Creative Commons Attribution License, Copyright 2022, The Authors, published by AAAS. (**d**) Vertical OECT-based inverter. (i) Schematic cross-section of the complementary inverter. (ii) The wiring diagram when the cofacial pair inverter amplifies the ECG voltage. (iii) Comparison of the recorded ECG signal between from the cofacial pair inverter and from medical electrodes using a benchtop digital multimeter [38]. Reproduced under the terms of the CC-BY 4.0 Creative Commons Attribution License, Copyright 2021, The Authors, published by AAAS.

In a wearable sensing circuit, the signal is typically amplified by transistors. The field effect transistor (FET) is a classical transistor exhibiting good performance in biosensors [25]. A basic insulation gate FET structure has three terminals, source, gate, and drain, in which the gate is separated from the body by an insulating SiO_2_ layer (Figure 3(ai)). A depletion layer is generated between the source and drain due to the migration of electrons and holes, and the magnitude of the drain current can be controlled by applying a voltage to the gate. In a wearable sensor, the region between the drain and source can act as a biometric element [25]. The shift in charge from changes in analyte concentration results in a change in gate voltage, and this difference results in a change in drain current, converting and amplifying chemical signals into electrical signals.

Wearable devices often require flexible, thin, and light components to fit the surface of the body. To meet these requirements, the thin film transistor (TFT) is developed by reducing the thickness of components in the FET. Increasing research has tapped its potential in flexible sensors [26,27]. Amongst various TFTs, organic thin film transistors (OTFT) and organic electrochemical transistors (OECT) are widely used.

The OTFT consists of three terminals, a dielectric layer, and an organic semiconductor layer. Compared with its inorganic structure metal oxide FET, using organic matter instead of metal oxide as the semiconductor layer can obtain higher flexibility and printability. By replacing the metal oxide dielectric with an organic dielectric, the OTFT is therefore flexible and printable. The connection between its terminal and organic semiconductor layer can be divided into top contact and bottom contact. Figure 3(aii) shows the OTFT structure with top contact, which has a larger charge contact area and smaller contact resistance compared to bottom contact. The result has easier access to high electrical conductivity, which is suitable for wearable sensors with low drive voltage.

To achieve higher transconductance and flexibility, the OECT is developed, whose nature is an electrolyte-gated FET. The electrolyte-gated FET regulates the charge accumulation in the electrolyte on the channel surface by the gate voltage, thus inducing a rapid but not strong charge migration within the channel. The OECT consists of metal terminals, organic semiconductor films, and electrolytes. In contrast to the electrolyte-gated FET, the gate voltage controls the electrolyte to inject ions into the organic semiconductor film directly, thereby changing the doping state of the organic semiconductor film and regulating the drain current (Figure 3(aiii)). Since the characteristic variation of the OECT occurs over the volume of the entire channel, opposed to the thin surfaces of conventional FETs, it can greatly regulate the drain current variation with a low gate voltage [28]. Although the flow of charge through the channel inevitably deteriorates the response time of the OECT, it gives satisfactory results when amplifying low frequency signals [28], such as electrochemical signals [29,30].

In general, transistor amplifiers have the advantages of low power consumption, small size, low power supply voltage requirements. What is more, they are resistant to physical shocks, exempt from preheating, and highly responsive to voltage change. Especially, the TFT has the advantages of high flexibility and processibility; the OTFT has flexible and printable characteristics, which make it suitable for sensors that fit on the surface of the skin; the OECT with liquid electrolyte has high transconductance and ultra-high flexibility at the expense of a reduced response speed, which makes it suitable for low frequency signal amplification. They are widely used in wearable devices, such as the portable electroencephalogram (EEG) measuring instrument [31], flexible electrocardiogram (ECG) patch [32], or ultra-conformal drawn-on-skin electronics [33]. To enhance the performance of transistors further, a lot of research (Table 1) has focused on the realization of lower power and higher CMRR amplification circuits.

#### 2.1.1. Energy Efficient Design

A large amount of energy is consumed by the amplification circuit in wearable sensors in order to enlarge the weak signal of interest [51]. Therefore, the amplification circuit studies of wearable sensors pay special attention to minimize power consumption while not affecting gain. 

Wearable sensors are often powered by tiny, low-voltage batteries. However, in practical applications such as location tracking, healthcare, environmental monitoring, etc., wearable devices are often needed to meet the demand for a certain amount of battery life. It challenges the ability of amplification circuits to operate at low voltages. Despite the good flexibility of OTFTs, the inherent low mobility and suboptimal switching properties of organic semiconductors make OTFTs require large drive voltages. However, the losses generated in the amplifier circuit increase as the drive voltage rises. Therefore, these losses can be reduced by lowering the drive voltage while keeping the gain constant. Exploring transistors with higher transconductance can effectively reduce the driving voltage and thus the power consumption. For example, Zhongzhong Luo et al. proposed an ultra-high gain OTFT that was optimized in terms of the material and process to obtain high transconductance [36]. This OTFT is made with Au as the electrode material, HZO/Al_2_O_3_ as the dielectric, and p-type 2,9-didecyl dinaphtho [2,3-b:2′,3′-f]thieno [3,2b]thiophene (C_10_-DNTT) as the channel material (Figure 3(bi)). Firstly, the HZO/Al_2_O_e_ gate dielectric is an extremely conductive material. Secondly, the C10-DNTT monolayer film made by a special shearing technique is highly crystalline, which will minimize the loss during the conductivity. Finally, the integrity of the monolayer organic film is preserved by the proposed solvent-free low-energy OTFT preparation process. These three points together result in the ultra-high electron mobility of the fabricated OTFT. Compared with the existing TFT fabrication process, the OTFT prepared by this process has ultra-high gain (Figure 3(bii)). Its ultra-thin, flexible nature makes it suitable for device arrays that fit on the surface of the skin (Figure 3(biii)) [36].

In addition to the OTFT based on the printing process, Ute Zschieschang et al. proposed an OTFT based on the electron beam lithography process [37]. This OTFT has three parts (Figure 3(ci)), a patterned aluminum gate, gold source, and gold drain; an aluminum oxide (AlO_x_) dielectric; and a phosphonic acid self-assembled monolayer (SAM). Among them, the SAM coating reduces the electron capture by the -OH group of the metal oxide layer, allowing higher electron mobility. Electrolithography is an advanced patterning technique, which was used for the first time in this study to prepare the OTFT on flexible substrates. The OTFT contact resistance, channel length, and gate-contact overlap obtained using this technique are extremely small. In addition, the results of previous studies [52] show that small values of these three metrics indicate that the obtained OTFT transmission is strong. The OTFT proposed in this study has an excellent on/off current ratio. The p-channel transistors have on/off current ratios as large as 4 × 10^9^ (Figure 3(cii)) and subthreshold swings as small as 70 mV/decade (Figure 3(ciii)), and the n-channel transistors have on/off ratios up to 10^8^ and subthreshold swings as low as 80 mV/decade. The high on/off current ratio indicates the high electron mobility of this OTFT, while the high subthreshold swing indicates that this OTFT switches rapidly between the on and light states. As of April 2022, it has the largest on/off current ratio among nanoscale OTFTs [37].

In addition to exploring high energy efficient transistors, a more compact transistor arrangement can also significantly reduce drive voltage and thus reduce energy loss. The ambipolar inverter composed of organic electrochemical transistor pairs with a coplanar vertical structure proposed by Reem B. Rashid et al. provides a new solution for low power consumption and weak physiological signal amplification [38]. These inverters convert low-voltage direct current into an elegant alternating current. Using a previously reported dry peel-off process [53], they deposited a first layer of Au, a parylene C (PaC) insulating layer, a second layer of Au, and a final insulating layer step by step on a glass slide. The ambipolar complementary inverter (Figure 3(ci)) has a vertical structure and a higher integration level per unit area than a planar structure. At the same time, the higher channel thickness makes it possible to control the drain current effectively with only a very small gate voltage; only 0.26 V can make about ten times the magnification (Figure 3(ciii)) of the ECG signal obtained from electrodes (Figure 3(cii)). Similar studies discussed the ability of the vertical OECT for low amplitude micro-organ signals and concluded that the OECT is well suited for amplifying tiny physiological signals [39].

Power consumption can also be reduced through current reuse technology [54]. Based on the fully differential current reuse amplifier design [55,56], Fatemeh Karami Horestani et al. proposed an ultra-low power amplifier suitable for collecting low-frequency biological signals [40]. In conventional structures, the input signal is injected into the gate of the negative metal oxide semiconductor (NMOS) transistor, and the positive metal oxide semiconductor (PMOS) transistor alone acts an active load. In the structure proposed in this study, two complementary transistors were simultaneously driven and operated as amplifiers. This design enables the amplifier to achieve two times higher transconductance at the same input voltage, meaning less power consumption for the same task. This technology is widely used in neural sensing [41], EEG monitoring [42], and self-powered devices [57].

#### 2.1.2. CMRR Enhancements

In the raw signals, the target signal is the differential mode while the noise signal consists of the differential mode part and common mode part. A high-performance amplifier can amplify more of the differential mode target signal in the raw signal to distinguish it from the noise, playing an important role in initial noise filtering. The CMRR is the ratio of the differential-mode gain to common-mode gain. The high CMRR represents a strong ability to suppress common mode noise. In wearable sensors, the amplification circuit faces common mode noises not only from the complex environments but also from its own mismatches.

**Figure 4:**
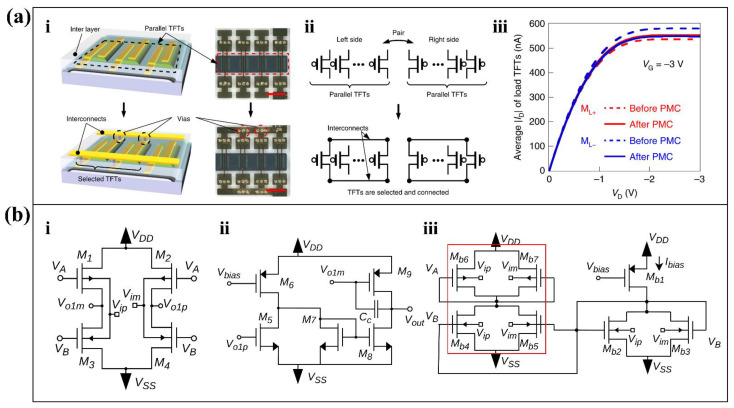
CMRR enhancement approaches of amplifiers. (**a**) PMC technology to reduce OTFT mismatch. (i) The parallel transistors are electrically isolated prior to connection, and electrical connection is provided by interconnecting metal lines through the insulating layer. (ii) The PMC process which is based on the compensation method in ref [43]. (iii) Output characteristics of two-sided OTFTs before and after PMC [58]. Reproduced with permission, Copyright 2019, Springer Nature. (**b**) CMFF compensated bias current design. (i) First stage of fully differential amplifier. (ii) Second stage differential to single ended amplifier. (iii) Schematic diagram of the replica bias circuit used to set the common mode current of the input stage [44]. Adapted under the terms of the CC-BY 4.0 Creative Commons Attribution License. Copyright 2020, The Authors, published by MDPI.

The noise of the transistor amplifier itself originates from the mismatch between the individual transistors. For example, the aforementioned OTFT has limitations in practical medical treatment and healthcare due to the electrical mismatch between OTFTs. Therefore, Masahiro Sugiyama et al. proposed an ultra-flexible organic differential amplifier [58] based on post-mismatch compensation (PMC) technology [43]. The differential pairs in the differential amplifier consist of parallel transistors. First, a top insulating layer which consists of polyethylene terephthalate produces electrical isolation of the paralleled transistors. Then, through-holes through the insulating layer and interconnecting metal wires provide electrical connections between the selected transistors (Figure 4(ai)). In the PMC process, the output characteristics of each parallel transistor are measured separately. Accordingly, the transistors are selected separately in the left and right groups to minimize the mismatch between the differential pairs (Figure 4(aii)). Finally, the results of Figure 4(aiii) show that the output characteristics of the OTFTs in the left differential pair (M_L+_) are much closer to those on the right (M_L-_).

Additionally, to meet the demands of low-voltage operation, traditional amplifier structures often have to make trade-offs between the number of differential pairs in the current mirror and the CMRR. A common-mode feed-forward (CMFF) technique [44] is proposed to solve this problem. The traditional bias current generator was removed, and the circuit shown in Figure 4(bi) and Figure 4(bii) was proposed as the input stage of the amplifier. One output of the first stage (Figure 4(bi)) is applied directly to the gate of the PMOS device, while the other produces the current mirror after NMOS co-sourcing, which is in the red box of Figure 4(biii). The second stage amplification circuit (Figure 4(bii)) changes the differential output of the first stage to a single-ended output to provide higher gain. Such compensation techniques improve the CMRR of the bias stage. According to its small signal model, the CMRR achieved by the proposed structure satisfies Equation (1).

(1) $$CMRR=\frac{1}{2}\times \frac{g_{mb1}+g_{mb3}}{g_{mb1}}\times \frac{g_{m1}}{g_{ds1}+g_{ds3}}\times \frac{g_{m5}g_{m8}+g_{m7}g_{m9}}{g_{m5}g_{m8}-g_{m7}g_{m9}}$$

This is the result when the approximation is generally assumed to be *g_m_ >> g_mb_* and *g_m_ >> g_ds_*, where *g_m_*, *g_mb_*, and *g_ds_*, represent the gate transconductance, bulk transconductance, and output conductance of the different transistors. Obviously, when *g_m5_* × *g_m8_* is close to *g_m7_* × *g_m9_*, the CMRR is close to infinity. The CMFF design makes the amplifier exhibit excellent common mode rejection, and it is suitable for wearable sensor amplification circuits.

In a multi-channel analog front end, assigning a dedicated reference to each channel is an effective solution to the CMRR degradation caused by impedance mismatches between differential inputs, but this increases power consumption significantly. Therefore, a 16-channel analog front-end application specific integrated circuit (AFE ASIC) using time division multiplexing (TDM) technology shares a second stage amplifier among eight channels, solving the impedance mismatch problem at low voltage [45]. Such a design is widely used in EEG and photoplethysmography (PPG) monitoring [46], infrared spectroscopy monitoring [47], and has the prospect of being applied to various biosensors [59]. The chopper stabilization technique is a traditional and effective amplifier linearization boosting technique that amplifies the signal by modulating it to a higher frequency free of noise, and then demodulates the amplified signal back to the baseband to reduce low-frequency noise [48]. This traditional technique was also optimized in recent studies. For example, a chopping control technology is proposed to solve the problem that the noise near the chopping frequency and its multiples is difficult to eliminate [49]. An excellent amplification circuit can suppress common-mode signals, but a single amplification circuit is not ideal for noise removal, which requires a filter circuit to further processing.

In fields such as motion capture, healthcare, and human-machine interface, motion artifacts are an almost unavoidable source of noise. Minimizing the effects of motion artifacts, especially in PPG monitoring [60], is critical to improving signal accuracy. To solve this problem, a direct-current-coupled (DC-coupled) structure is often used in amplifiers, which connect the input directly to the gate of the transistor to achieve an input impedance far exceeding that of AC-coupled structure. However, the capacitance mismatch of the conventional three-amplifier DC-coupled circuit has a negative impact on the CMRR of the amplification. Therefore, a fully differential difference amplifier (FDDA) which can replace the three-amplifier topology [50] was proposed to improve the direct-current-coupled amplifiers’ CMRR. The proposed circuit employs cascaded current mirrors to increase impedance to suppress tail current mismatch and larger size transistors to reduce transistor mismatch. In general, a larger transistor size will occupy the space of the radio capacitance and lead to the deterioration of the CMRR. This study proposes a parasitic capacitance reuse technique to mitigate this deterioration by using parasitic capacitance to share a portion of the specific capacitance. A similar design [61] compresses the three operational amplifiers’ (OPAMP) circuit and replaces it with two single-stage operational transconductance amplifiers (OTA), realizing a differential difference amplifier (DDA) circuit. The bias current of the OTA is shared in the superposition method, and the self-stabilizing current is distributed to the common mode feedback (CMFB) circuit through the MOS pseudo-resistor, thereby improving the CMRR of the circuit.

### 2.2. Filtering

Wearable sensors are often adversely affected by different sources of noise. For example, when performing ECG signal acquisition, they are not only subject to motion artifact interference and myoelectric clutter interference, but also to power line interference (such as 50 Hz or 60 Hz noise from the power line) [62]. In order to remove the noise and retain the signal of interest to the greatest extent [63], the filter needs to be selected according to the characteristics of the noise and the requirements for the filtering result.

According to the form of the target signal that can be processed by the filter, filters can be divided into analog filters and digital filters. Using the characteristics of inductors to pass through high-frequency signals and capacitors to block low-frequency signals, an easy-to-implement analog filter can be made. By adjusting the size and layout of the inductor and capacitor, the analog filter can only allow signals in a certain frequency range to pass through to achieve the effect of filtering out spurious waves. The digital filter is an algorithm or device composed of an adder, a digital multiplier, and a delay unit. Unlike analog filters, digital filters do not use the electrical characteristics of electronic components for filtering, but modify the digital code of the input discrete signal for the purpose of changing the frequency of the signal. Compared with analog filters, digital filters are smaller in size and more complex in design. In addition, its filtering efficiency would not be affected by the aging of the circuit, so its filtering accuracy is far higher than the corresponding analog filter [24].

Many types of filters have been proposed that can perform segmentation with different characteristics for target signals in various frequency bands. Depending on the parameters of the filters, each filter has different orders. The characteristics of the filter become apparent as the order increases, but the difficulty of implementation increases as well.

#### 2.2.1. Filter Type

According to the frequency response function achieved by the filter, the common filters in wearable sensors can be divided into three types: Butterworth filter, Chebyshev filter, and Elliptic filter, which can segment the target signal with different characteristics. It is worth noting that the three types are not fixed analog or digital filters, and the conversion between analog filters and the corresponding digital filters can be achieved through algorithms (such as the impulse invariance method or bilinear transform) [64].

##### Butterworth Filter

**Figure 5:**
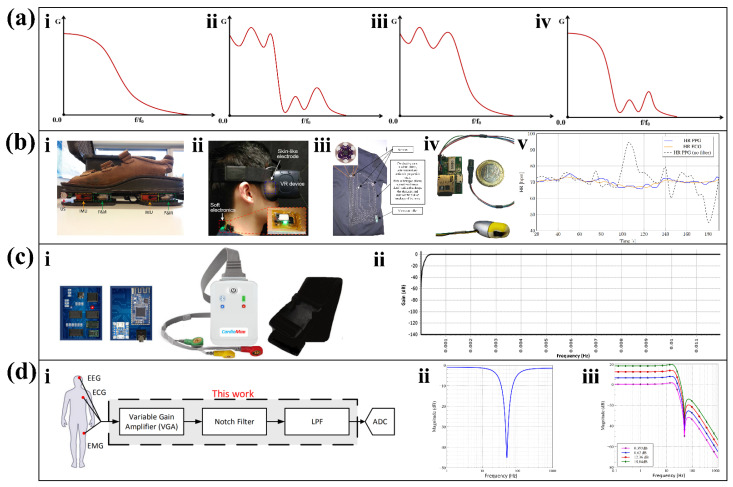
Characteristics and applications of three different types of filters. (**a**) Filter frequencies of Butterworth filter (i), Elliptic filter (ii), Chebyshev I filter (iii), and Chebyshev II filter (iv). (**b**) Wearable devices with different orders of Butterworth filters. (i) Gait and dynamic balance sensors using second-order Butterworth filters [66]. Reproduced under the terms of the CC-BY 4.0 Creative Commons Attribution License. Copyright 2019, The Authors, published by IEEE. (ii) Eye-tracking device with third-order Butterworth filter [67]. Reproduced under the terms of the CC-BY 4.0 Creative Commons Attribution License. Copyright 2020, The Authors, published by AAAS. (iii) Physiological monitoring clothing for firefighters using a fourth-order Butterworth filter [68]. Reproduced with permission. Copyright 2018, IEEE. (iv) In-ear PPG monitoring device with a fourth-order Butterworth filter. (v) Heart rate estimation during slow walking [69]. Reproduced with permission. Copyright 2020, IEEE. (**c**) An ambulatory ECG monitoring system. (i) Front view of the electrocardiograph. ii. The response of Chebyshev filter at high pass filtering [70]. Reproduced under the terms of the CC-BY 4.0 Creative Commons Attribution License. Copyright 2021, The Authors, published by MDPI. (**d**) Biopotential signal detection front-end with Elliptic filter. The block diagram (i), magnitude response of the proposed notch filter (ii), and low pass filter (iii) [71]. Reproduced with permission, Copyright 2020, IEEE.

The characteristics of the output frequency band of the Butterworth filter are shown in Figure 5(ai). As one of the most mainstream filters, the Butterworth filter has the characteristic that the frequency has good stability both inside and outside the pass frequency range, and the frequency band is maximally flat in the pass band. However, Butterworth filters have the disadvantage of a slow descent in the stop band, resulting in a long equivalent transition band. If the signal of interest happens to be within the transition band, it is prone to distortion. This disadvantage can be overcome as the filter order increases. The decay of the resistance band accelerates with increasing order, resulting in more accurate processing results. Based on the above characteristics, the Butterworth filter is suitable for cases where the passband and stopband ripples are small, and the requirements for the transition band signal are low [65]. The frequency response of the Butterworth filter satisfies Equation (2).

(2) $$G_{n}\left(\omega \right)=\frac{1}{\sqrt{1+\omega^{2n}}}\;$$

where ***ω*** is the corner frequency of the filter, and *n* is the order of the filter. Depending on the accuracy requirements of the desired results, developers can choose different orders of Butterworth filters.

When choosing the Butterworth filter order, accuracy and complexity should be balanced. For example, in a wearable foot sensor for gait and dynamic balance sensing (Figure 5(bi)), the designer uses a second-order Butterworth filter to finish the low-pass filtering task [66]. In a wearable device for real-time detection of eye vergence in a virtual reality (Figure 5(bii)), a third-order Butterworth filter is used for band-pass filtering [67]. In a wearable sensing device (Figure 5(biii)) that monitors the physiological signs of firefighters in real time, a fourth-order Butterworth filter is used for low-pass filtering of the accelerometer [68]. In the wearable gait evaluation, after considering the complexity and steepness (rate of descent) of the design, a fourth-order Butterworth filter is chosen by compromise. Combined with the filtered output frequency band, the characteristics of the flat passband of the Butterworth filter can be further recognized. When performing in-ear continuous PPG monitoring, a fourth-order Butterworth low-pass filter with a cutoff frequency of 10 Hz was selected as the low-pass filter [69]. Figure 5(biv) shows the appearance of the in-ear PPG monitor, and Figure 5(bv) shows the beats per minute under normal walking. In the period from 70 s to 170 s, several abnormal heartbeat frequencies are higher than the actual value but are well suppressed after filtering. The fourth-order Butterworth low-pass filter used by the device shows a good filtering ability on the high-frequency noise. The results of these studies show that Butterworth filters can effectively filter out signals outside the passband and flatten the frequency.

##### Chebyshev Filter

Compared with the Butterworth filter, the same order Chebyshev filter drops faster in the stop band, but the response in the pass band fluctuates. Chebyshev filters are further divided into two types: Chebyshev I filters which have equal ripple in the pass band and flat in the stop band; Chebyshev II filters which have flat in the pass band and equal ripple in the stop band. The characteristics of the output frequency band of the Chebyshev I filter and Chebyshev II filter are shown in Figure 5(aiii) and Figure 5(aiv), respectively. The frequency response of the Chebyshev I filter satisfies the Equation (3), and the frequency response of the Chebyshev type II filter satisfies the Equation (4).

(3) $$\;G_{n}\left(\omega \right)=\frac{1}{\sqrt{1+\varepsilon^{2}T_{n}{}^{2}\left(\omega_{0}/\omega \right)}}$$

(4) $$G_{n}\left(\omega \right)=\frac{1}{\sqrt{1+\frac{1}{\varepsilon^{2}T_{n}{}^{2}\left(\omega_{0}/\omega \right)}\;\;}}$$

(5) $$T_{n}\left(\Omega \right)=\cos\left(n*arccosx\right);0\leq x\leq 1$$

Among them, ***ω*** is the angular frequency of the filter, and *n* is the order of the filter, satisfying Equation (5). The *x* indicates ***ω_0/_ω***.

As reflected in an IoT-assisted ambulatory ECG monitoring system with arrhythmia detection (Figure 5(ci)) [70], the rapid drop in the stopband is an advantage of the Chebyshev filter in general. This study proposes a wearable arrhythmia monitor that sets up four-stage filtering in the first round of filtering in which three stages use Chebyshev II filters. In the first stage, it uses a second-order Chebyshev high-pass filter with a cutoff frequency of 0.5 Hz. In the second and third stages, a second-order band stop Chebyshev II filter that provides a 10 Hz notch at 50 Hz is used. Figure 5(cii) shows the programmable embedded system-on-chip (PSoC) response of the Chebyshev II filter through the first stage. This finding shows that the frequency profile decreases rapidly from 0.5 Hz to 0 Hz, indicating that the second-order Chebyshev high-pass filter has a good filtering effect in the first stage. However, sometimes too rapid a drop can also have an adverse effect on the results. For example, the rapid drop in the stopband of a Chebyshev I filter gives it a narrower transition band than a Chebyshev II filter, which can adversely affect its ability to filter out anomalously correlated signals [72].

##### Elliptic Filter

Elliptic filters, also known as Cauer filters, are equiripple in both passband and stopband; from Figure 5(aii), we can see these characteristics. It is different from the Butterworth filter with a flat passband and an equal ripple in the stopband, and the Chebyshev I filter with an equal ripple in the passband and a flat stopband. Comparing with the filters mentioned before, at the same order, Elliptic filters have the smallest passband and stopband fluctuations, as well as the narrowest transition band. Elliptic filters require only a lower order to achieve the same accuracy as Butterworth and Chebyshev filters. The frequency response of the Elliptic filter satisfies Equation (6).

(6) $$G_{n}\left(\omega \right)=\frac{1}{\sqrt{1+\epsilon^{2}R_{n}^{2}\left(\omega \right)}}$$

where ***ω*** is the angular frequency of the filter; *n* is the order of the filter; and *R_n_* is the Jacobian Elliptic function of *n*.

Elliptic filters have the significant features of a narrow transition band and fast attenuation. Accordingly, Maha S. Diab et al. adopted Elliptic filters in a general front-end for biopotential signal detection [71]. The front end can be used for EEG, ECG, and EMG signal acquisition, which is further processed by a filter after passing through a variable gain amplifier (VGA) (Figure 5(di)). In this research, a second-order trap elliptic filter was used to reduce 50 Hz power line interference, and a fourth-order low-pass elliptic filter was used to filter out high frequency noise above 24 Hz. The results show that the designed filter can accurately reduce power line interference at 50 Hz (Figure 5(dii)), and the low-pass filter shows fast attenuation in the operating range (Figure 5(diii)).

The simplicity of the structure is an essential advantage of Elliptic filters. In a study [73] which needs an active bandpass filter for the fifth-order OTA, the stopband frequencies of different orders of the Butterworth filter, Chebyshev filter, and Elliptic filter were measured. When reaching the same stopband frequency, the order required by the Elliptic filter is much smaller than that of the Butterworth filter and slightly smaller than that of the Chebyshev filter. Therefore, this study selects the Elliptic filter for further design in order to minimize power consumption.

#### 2.2.2. Applications and Innovation of Filters

Depending on the differences in operating characteristics, each structure of the filter is suitable for processing specific types of signals. Meanwhile, the proposed new filtering strategies provide solutions for more accurate and effective noise filtering (Table 2).

In terms of popularity, Butterworth filters have stronger generalizability compared to Chebyshev filters and Elliptic filters. In the field of motion recognition, Butterworth filters can not only be used with accelerometers and EMG sensors to complete a motion evaluation of the spine [74] and gait [75], but also can be combined with machine vision to recognize human behavior [76]. In addition, Butterworth filters can be used to deal with low frequency noise in ECG [77] and high frequency noise in EEG [77]. The possible reason is that the flat passband of the Butterworth filter highlights the transient changes of the EMG, but the longer transition band of the Butterworth filter blurs the details of the ECG and EEG. In general, Butterworth filters are suitable for processing signals that require low stopband decay rates and are particularly suitable for myoelectric signals.

**Figure 6:**
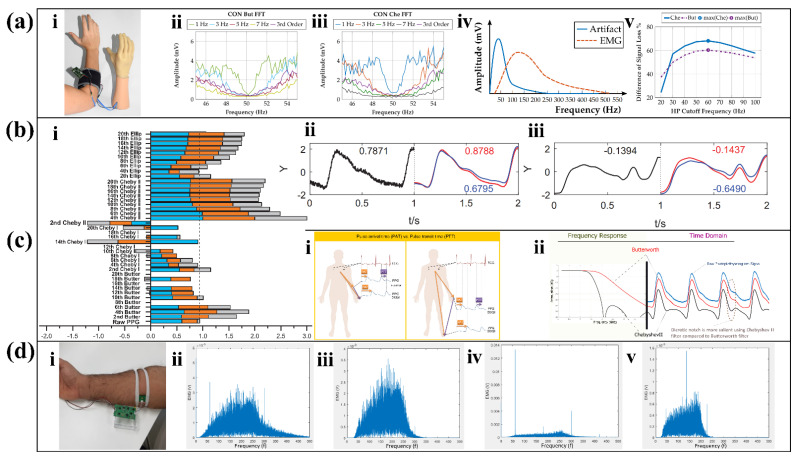
Application and innovation of filters. (**a**) Insulated EMG sensing. (i) The myoelectric sensor is fixed on the human forearm through a cuff. Butterworth-filtered (ii) and Chebyshev-filtered (iii) signal at contracted muscle. (iv) Qualitative sketches of systolic EMG and typical motion artifacts in the lower range. (v) Difference between artifact and EMG signal losses [78]. Reproduced under the terms of the CC-BY 4.0 Creative Commons Attribution License. Copyright 2019, The Authors, published by MDPI. (**b**) Optimal filter selection in PPG. (i) SQI for all types of filters. The excellent (ii) and unfit (iii) PPG signal processing comparison between Butterworth filter (blue) and Chebyshev filter (red) [79]. Reproduced under the terms of the CC-BY 4.0 Creative Commons Attribution License. Copyright 2018, The Authors, published by Springer Nature. (**c**) The use of PPG for assessing hypertension. Difference between PAT and PTT (i) and the filters’ impact on PPG morphology (ii) [90]. Adapted under the terms of the CC-BY 4.0 Creative Commons Attribution License. Copyright 2021, The Authors, published by Springer Nature. (**d**) EMG signal processing instrument (i) with adaptive filter. Comparison of EMG signal before (ii) and after (iii) processing and noisy EMG signal before (iv) and after processing (v) [85]. Reproduced with permission, Copyright 2019, IEEE.

For overlapping parts of the signal of interest and noise, the Chebyshev filter has a stronger decoupling capability than the Butterworth filter at the same order. Theresa Roland et al. screened them when designing an ultra-low-power filter module [78] for isolated EMG sensors (Figure 6(ai)). EMG signals are susceptible to power line interference and motion artifacts. Since the common power line frequency is 50 Hz, the study compared the processing capabilities of the two filters for EMG signals around 50 Hz. Taking a cutoff frequency of 5 Hz as an example, the Butterworth filter performs better as it exhibits less overshoot (Figure 6(aii)). The results of the Chebyshev filter are characterized by high gain at the edge of the passband (Figure 6(aiii)), which adversely affects power line frequency fluctuations. The reason for this result is that the passband and transition band of the Butterworth filter are flat. Since the low-frequency motion artifact signal is close to the EMG signal during contraction (Figure 6(aiv)), the filter needs to preserve the EMG signal while filtering out coincident motion artifacts as much as possible. At this time, the high-pass Chebyshev filter can maximize the differentiation of these two types of signals (Figure 6(av)).

The Chebyshev filter is also the most advantageous filter for PPG measurements where high accuracy is often required for PPG signals to obtain the pulse arrival time (PAT) and pulse transmission time (PTT) with the reference value (Figure 6(ci)). Yongbo Liang et al. explored the most suitable filters for PPG signals and compared the three filters mentioned in this paper [79]. First, by comparing the filtered signal quality index (SQI), an important indicator for evaluating filter capacity, it found that Chebyshev II filter and Butterworth filter have obvious advantages in normalization processing (Figure 6(bi)). Then, the study compared the effectiveness of the Chebyshev type II filter and Butterworth filter for processing different qualities of the raw signal. The conclusion shows that the Chebyshev II filter can improve the quality of the signal more effectively than other types, regardless of whether it is a high-quality signal (Figure 6(bii)) or an unsatisfactory signal (Figure 6(biii)). Among them, the four order Chebyshev II filter has the highest quality. Since the Butterworth filtering result is flat and has a long transmit band, it weakens the dicrotic notch in the PPG signal (Figure 6(cii)), which is an important indicator in PPG measurements [91]. At present, many studies affirmed the ability of the Chebyshev II filter to filter high frequency noise in PPG [80,81].

In the field of wearable sensors, Elliptic filters are not as popular as the other two types of filters, and most studies on Elliptic filters focus on its ability to handle ECG signals. In 2018, Navdeep Prashar et al. proposed a removal of electromyography noise from ECG for high-performance biomedical systems [82]. The researchers compared the Butterworth filter, the Chebyshev I filter, and the Elliptic filter in terms of noise filtering in the high-frequency part of the ECG, and the result is shown in Table 3. At the same time, this article also lists similar research work on noise removal in ECG signals, and compares the filtering effect of the wearable device with their effect. The final conclusion of this article is that the signal-to-noise ratio (SNR) of the Elliptical filter is improved the most, while the power spectral density (PSD) of the Elliptical filter is reduced the most, so the Elliptical filter has the best effect of low-pass filtering among all the filters in the case of high-frequency noise generated. Moreover, because the Elliptical filter transition band is so narrow, ECG sensors often use it precisely to eliminate 50 Hz or 60 Hz power line interference [83,84].

However, due to the complex and changeable environment in which wearable sensors are located, noise often exists in multiple frequencies and changes constantly, and filters with fixed coefficients are difficult to cope with such a complex environment. The adaptive filter structure for EMG sensors (Figure 6(di)) proposed by Muhammad Zaka Jamal et al. can deal with this problem [85]. This structure adopts an adaptive weighting algorithm, which continuously adjusts the weight of the filter coefficients according to the result of the feedback after filtering to remove different noises. Since the power line interference in the United States is 60 Hz, when the human body touches the surface of the power supply, battery, etc., the EMG signal will be interfered by 60 Hz and its harmonics (Figure 6(dii)). By using adaptive filters, the interference signals concentrated at 60 Hz and 300 Hz are effectively suppressed (Figure 6(diii)). For severely disturbed EMG signals (Figure 6(div)), the filter can also effectively filter out the main noise components at 60 Hz, 180 Hz, and 300 Hz (Figure 6(dv)), and has a higher SNR than common instrument circuits.

Furthermore, many ingenious algorithms have been proposed to solve the problem of unsatisfactory filtering accuracy, and the particle filter is a typical example. Based on statistics and probability, it treats the raw signal as a set of particles (also called samples), and assigns weights to each particle and predicts its changes [92]. The accuracy of particle filters decays rapidly with increasing dimensionality, and the particle filter codes working in low-dimensional conditions have a simple structure and scalability [86]. These features make them suitable for wearable positioning [86,87]. However, highly integrated sensors are becoming mainstream, especially in the fields of emotion recognition and motion capture. They require the fusion of multiple signals and the minimization of complex noise interference. Sensor fusion techniques [93] using different filtering algorithms, such as complementary filtering (CF), extended Kalman filtering (EKF), and unscented Kalman filtering (UKF), can centrally process data from multiple sensors. The algorithm of CF sensor fusion technology is simple and reliable [94], without a divergence problem, but it can only be used when the sensor frequency characteristics are complementary [95]. Kalman filters are more efficient and accurate, and UKFs are particularly suitable for heavily nonlinear signal processing, such as accurate wearable heading tracking [88] and human joint angle estimation [89].

## 3. Analog-to-Digital Conversion

The physiological signals (such as bioelectricity, pulse, respiratory rate, etc.) are all collected by the wearable sensor in the form of analog signals. However, the analog signals cannot be directly recognized by the computer. Therefore, an ADC is required to convert continuous analog signals into discrete digital signals. A high-performance ADC can accomplish this process accurately and minimize the additional noise introduced. In wearable sensors, the performance of the ADC will be evaluated in terms of linearity and energy efficiency. We will first briefly introduce five mainstream ADCs and then highlight the strategies of enhancing linearity and energy efficiency.

### 3.1. ADC Types

According to the device structure, the mainstream ADCs can be typed as flash ADC, pipeline ADC, SAR ADC, dual slope ADC, and sigma-delta ADC.

#### 3.1.1. Flash ADC

**Figure 7:**
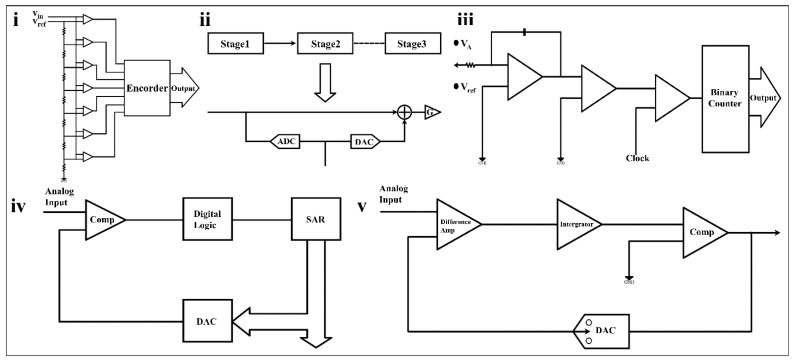
Block diagrams of five types of ADCs. (i) Flash ADC [96]. (ii) Pipelined ADC. (iii) Dual slope ADC. (iv) N-order SAR ADC [96]. Reproduced with permission. Copyright 2016, IEEE (v) Sigma-delta ADC.

Among these five types of ADCs, the flash ADC has the fastest operating speed. It adopts a linear voltage ladder structure as shown in Figure 7i. During the operation of the flash ADC, each node is connected to 2N-1 comparators to compare the input voltage with a continuous reference voltage. Multiple comparators need to work in parallel, resulting in fast operation speed and huge power consumption.

#### 3.1.2. Pipeline ADC

The pipelined ADC has a better balance between performance and power consumption than the flash ADC. A pipelined ADC consists of multiple stages, each containing a sub-ADC, a digital-to-analog converter (DAC), as shown in Figure 7ii. The data quantized by the ADC is fed to the DAC for processing, and the analog input is then subtracted from the sampled input, resulting in a residual voltage that goes through the accumulator (G) to the next stage. The pipeline ADC has a high conversion rate and low power consumption, which is widely used in high-speed and high-precision fields such as wireless communication and digital video [97]. However, its complex bias structure and high circuit process requirements, as well as the delay in the signal traversal through the digital circuit [98], make it unsuitable for simple, sensitive wearable sensors.

#### 3.1.3. Dual Slope ADC

The dual-slope ADC has the advantages of high accuracy and low power consumption, but its operating speed is much lower than that of SAR ADCs [99]. It consists of an integrator, comparator, timer, and binary counter, as shown in Figure 7iv. When the dual slope ADC starts working, the binary counter, timer, and integrator import an unknown analog voltage (V_A_), and the comparator output is positive. When the counter reaches a fixed count, the timer is cleared; a negative reference voltage (V_ref_) is introduced; and the timing starts again. When the comparator output is negative, V_A_ can be digitized from the reference voltage value and two timings. Dual slope ADCs do not require DACs, nor do they require as many comparators as other ADCs, which helps simplify the design [100].

The dual-slope ADC owns high conversion accuracy and can achieve a high-quality analog-to-digital conversion by using a long conversion time under the condition of a low sampling rate. This characteristic makes it suitable for target signals with small bandwidths. As in PPG monitoring, dual-slope light-to-digital converters (LDC) enable high-precision conversion at low sampling rates [101], which require very low power consumption to complete the task.

#### 3.1.4. SAR ADC

The SAR ADC is one of the ADCs that this paper will focus on, and it is the most common ADC in the analog-to-digital conversion of wearable sensors. It consists of SAR, DAC, and voltage comparators, as shown in Figure 7iii. The resolution of the SAR ADC is generally between 8 bits and 16 bits. Its sampling rate is generally below 5 MS/s, which is not suitable for application scenarios requiring high precision and a high sampling rate. However, SAR ADC has significantly low energy consumption and can be dynamically tuned compared to the other four types of ADCs. At the same time, it requires far less of a silicon area than a pipelined ADC to achieve its equivalent capability. Thus, it is considered to be the most suitable device for biomedical applications [102].

The superiority of SAR ADC dynamic regulation deserves to be further emphasized. Faced with different usage scenarios of PPG monitoring [103], the ADC can run at 45 nA at a 250 S/s sampling rate or 90 nA at a 500 S/s sampling rate, which means it can not only meet high-load working conditions but also guarantee battery life under low-load conditions. As another example, the SAR ADC module adopted in the gesture recognition system [104] has rich tunable levels. It allows easy adjustment of the sample rate by dynamic voltage and has different resolution options to meet various conversion requirements. Among such devices, the SAR ADC exhibits its flexibility with multi-level adjustability and excellent power control ability. At present, a lot of research efforts are dedicated to provide low-power and high-performance ADC for wearable sensors.

#### 3.1.5. Sigma-Delta ADC

The sigma-delta ADC is extremely accurate, with conversion rates between SAR ADCs and dual-slope ADCs and higher power consumption than the SAR ADC [105]. It consists of a differential amplifier, an integrator, and a comparator (Figure 7v), operated at a rate well above the bandwidth of the analog signal to provide oversampling. The analog signal is differentially compared with the feedback signal, and the result is fed into the integrator. Next, the result of the integrator is received by the comparator. The comparator will generate a feedback signal and send it to the differential amplifier, and simultaneously output the digital result.

The features of low speed and high accuracy make the sigma-delta ADC suitable for sensors with a narrow target signal bandwidth and high precision requirements, such as EEG monitoring [106] and knee joint activity monitoring [107]. In addition, it can be combined with the SAR ADC to produce an ADC structure with superior performance, which will be introduced later.

### 3.2. ADC Optimization Strategy

Since the SAR ADC and sigma-delta ADC are widely used in the field of wearable sensors, this paper focuses on their optimization strategies (Table 4). These strategies can be divided into two aspects. The first is how to maximize the linearity of the ADC without sacrificing the sampling rate and resolution. Linearity is an important indicator to describe the static characteristics of the sensor which shows similarity between actual and ideal results obtained by the ADC. The spurious-free dynamic range (SFDR) and signal-to-noise distortion ratio (SNDR) are commonly used to reflect the linearity of an ADC. The SFDR is the ratio of the maximum signal component to the next largest noise component, and the SNDR is the ratio of all undesirable frequency components to the input frequency. The second is to minimize power consumption without compromising its performance as much as possible, which is similar to the efficient design of the amplification circuit mentioned earlier.

#### 3.2.1. Linearity Enhancement

The ADC, as an active unit to undertake the task of signal conditioning and connect the DT, is vulnerable to various adverse effects such as power supply noise, capacitor mismatch, current leakage, and so on. The negative effects introduced by these factors make it difficult to achieve high-precision versions of conventional ADCs without or with less use of large calibration circuits and large cell capacitors. Low-precision ADCs, on the other hand, are insufficient for applications such as emotion recognition [129] and human-machine interfaces [130]. Therefore, many studies have explored ways to improve the linearity of the ADC without increasing the circuit area.

In conventional SAR ADC structures, the leakage current that will inevitably be generated by the voltage difference between the base and collector of the transistor (V_M_ and V_CTOP_) is an important issue that deteriorates the linearity of the ADC. To solve this problem, a voltage-optimized design [108] added a mini-c-DAC to assist the main DAC to complete the conversion process. The presence of the mini-c-DAC reduces the voltage at V_M_ to bring the two voltages closer, thereby significantly reducing the leakage current. In a study to eliminate spectral spurs [109], a complete scheme to improve ADC linearity with an emphasis on DAC optimization was proposed. First, the study proposes a tracking algorithm, which improves the SNR and achieves a stable error correction during high-speed operation by repeating eight least significant bit (LSB) comparisons and averaging the results. Then, the DAC is filtered to reduce the noise generated by the comparison stage. Finally, in order to reduce the error caused by the mismatch of the DAC capacitors, this research proposes a Noise-Shaped-Uniform (NSU) dither jitter injection method based on the ADC dithering method [131]. After this series of optimized designs, the proposed ADC achieves a SNDR of 71 dB and a SFDR of 85 dB.

**Figure 8:**
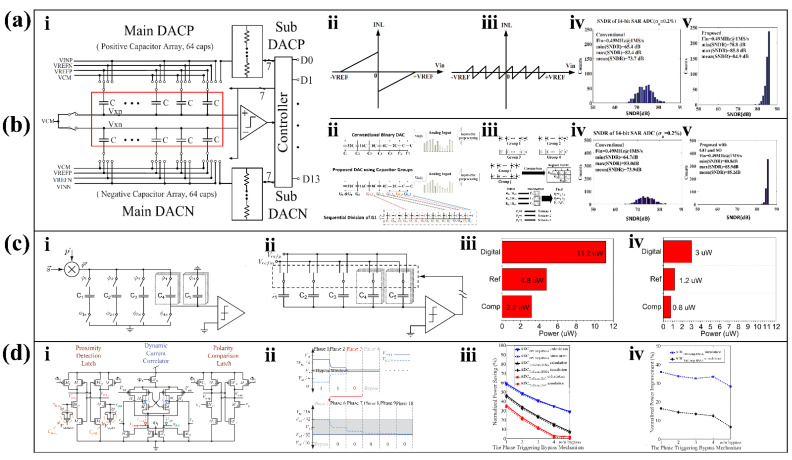
ADC linearity enhancement and energy efficient design. (**a**) Capacitor mismatch solution with a SAR ADC structure which has 14-bit capacitor and resistor combination (i) that break the original INL (ii) for a smaller margin of error (iii). In addition, the improvement in SNDR (iv) and SFDR (v) it brings is considerable [110]. Reproduced with permission, Copyright 2018, The Authors, published by IEEE. (**b**) On the basis of (**a**), the capacitor allocation is improved (ii), and the optimization strategy is added (iii). In addition, it shows more ideal SNDR (iv) and SFDR (v) [111]. Reproduced under the terms of the CC-BY 4.0 Creative Commons Attribution License, Copyright 2018, The Authors, published by IEEE. (**c**) Special CS design. DAC array with 12-bit CS-SAR ADC, with sampling cycles from φ1 to φ4 (i) and quantization cycle φ5 (ii), achieved the power consumption; (iv) is much lower than that of Nyquist mode (iii) [116]. Reproduced with permission, Copyright 2017, IEEE. (**d**) SAR ADC structure (i) with dynamic comparator, bypass-switching process (ii), and code recovery operation. Both bypass-switching technology (iii) and dynamic comparison technology (iv) can significantly improve energy efficiency [117]. Reproduced with permission, Copyright 2018, IEEE.

What is more, the optimized capacitive arrangement is also an important research direction in the improvement of ADC linearity. Figure 8(ai) shows a 14-bit SAR ADC structure consisting of a combination of capacitors and resistors. This multi-capacitor, high-resolution ADC is prone to accumulating errors from capacitor mismatch, which affects the linearity of the ADC. Therefore, a study applied a capacitor reorganization method by introducing 64 new capacitors for screening [110]. The proposed capacitive array can change the original integral nonlinear results. Compared with the traditional structure (Figure 8(aii)), the integral non-linearity (INL) range achieved by the new array is greatly reduced (Figure 8(aiii)). Taking the capacitance mismatch of σ_u_ = 2% as an example, the SNDR of the proposed SAR ADC (Figure 8(av)) is improved compared with the traditional structure (Figure 8(aiv)) under various conditions.

Algorithm optimization is the most common way to improve ADC linearization. Since the method mentioned above [110] introduced 64 additional capacitors, some of researchers achieved better results in another study by implementing the minimum error algorithm [111] instead of introducing additional capacitors. First, the original capacitor array was disassembled and divided into four groups to form the newly proposed structure (Figure 8(bii)). Next, three different alternative calculation strategies for minimizing error terms are preset. Finally, four capacitor groups obtain the minimum error term after completing six comparisons and “XNOR” operations (Figure 8(biii)). Taking the capacitance mismatch of σ_u_ = 2% as an example, the SNDR of the proposed SAR ADC (Figure 8(bv)) is improved compared with the traditional structure (Figure 8(biv)) under various conditions, which is also superior than the structure [110] mentioned before.

In addition to the above mainstream methods, there are many other ways to improve the linearity of the ADC. In order to ensure the linearity of the sampling switch, it is important that the on resistance of the transistor is stable across the voltages. Studies show that transistors with larger aspect ratios have less resistance change in the face of voltage changes and are more conducive to improving the linearity of the ADC [132]. Mohammad Tohidi et al. proposed a neural-signal-specific ADC suitable for wearable neural signal monitoring [112]. The ADC proposed in this study can discriminate the target signal from clutter and perform a higher precision analog-to-digital conversion of the target signal to achieve the effect of noise reduction. We found that not much research has focused on improving the linearity of sigma-delta ADCs directly, but combining sigma-delta modulation with SAR ADCs can have the effect of improving linearity. Since conventional SAR ADCs require complex on-chip linearization techniques in order to achieve resolutions above 12 bits, Ahmad AlMarashli et al. applied sigma-delta modulation to the DAC unit of conventional SAR ADCs [113]. Converting a full-resolution (N-bit) digital word in SAR logic to a coarsely quantized output is accomplished with high precision, taking advantage of its simplicity and insensitivity to capacitance mismatch. After this research, the researchers proposed a similar design in 2017, using the inherent linearity of the sigma-delta DAC to replace the traditional DAC to complete the calibration step in the SAR ADC [114]. In a high-resolution ADC design for biosensor arrays, the SAR ADC is used to encode sigma-delta modulation, which combines SAR ADCs’ fast rate and sigma-delta modulation’s high precision [115]. In conclusion, the combination of sigma-delta modulation and SAR ADC is an ADC linearity optimization strategy that can take into account the conversion rate, power consumption, and high resolution.

#### 3.2.2. Energy Efficient Design

Although the SAR ADC has the feature of energy-saving, it does not stop scholars from exploring more energy-efficient ADC circuits. The energy loss of the ADC mainly comes from the switch discharge and capacitor discharge. Therefore, existing research efforts have focused on reducing the number of capacitors by circuit rationalization or minimizing the discharge frequency of existing switches and capacitors.

Conventional sampling protocols based on Nyquist’s theorem have high requirements for data acquisition rates, which must be at least twice the signal bandwidth. Part of the physiological signals collected by wearable sensors are sparse, and over-sampling due to Nyquist’s theorem consumes additional energy. Compressive sensing (CS) was proposed to recover sparse signals from measurements well below the Nyquist rate accurately. Ordinary CS encoders require the OTA for continuous integration or low-pass filtering, which bring additional energy consumption. Wenjuan Guo et al. proposed a CS-SAR ADC structure that uses a fully passive switched capacitor circuit without the OTA to save energy, as shown in Figure 8(ci) [116]. Among these capacitors, C_1_ to C_4_ constitute the most significant bit (MSB), and C_5_ constitutes the LSB. During operation, φ1 to φ4 are sampling stages (Figure 8(ci)), and φ5 is the quantization stage (Figure 8(cii)). Four equally weighted samples take place at φ1 to φ4, each sampling only a quarter of the total capacitance, and then φ5 quantifies the average result of the four samples. Such a design sacrifices a certain operating speed, but can make the proposed input buffer power (Figure 8(civ)) as low as one-fourth of the traditional structure (Figure 8(ciii)).

Additionally, for sparse ECG signals, Tzu-Yun Wang et al. proposed a bypass-switched SAR (BSSA) ADC with a dynamic proximity comparator [117]. Most of the sampled ECG signals are close to the common-mode level. If the SAR ADC can detect whether the sampled signal is close to the common mode level, and skip several previous MSB conversion steps, it can effectively save switching energy. First, it determines whether the input signal is within the bypass window by means of a dynamic proximity comparator with a current correlator. Second, the size of the bypass window can be changed by adjusting the charging speed of the V_bump_ and V_diff_ nodes (Figure 8(di)). Next, the input signal passes through the bypass switch. In the first four conversion stages, as long as the comparator input voltage falls within the bypass window, the conversion process jumps directly to the sixth stage and skips the intermediate stages (Figure 8(dii)). In order to obtain the complete binary code after skipping the MSB step, four full adders are used to restore the final output bits of the first five MSBs. The proposed BSSA logic provides more than a 10% energy saving improvement (Figure 8(diii)), while the dynamic proximity comparator further improves the energy saving by 20% (Figure 8(div)).

Traditional SAR ADCs use the MSB priority during the DAC, and such N-bit ADCs need to perform N-time comparisons to achieve conversion. For weak signals such as cardinal signals, an algorithm based on a LSB priority was proposed [118]. It achieves N-bit effects at less than N times calculations by predicting signal change trends. On its basis, the common voltage (V_cm_) was introduced [133] as an improvement, which halves the number of capacitors. For frequently triggered, slowly changing neural signals, a six-bit SAR ADC can be used to replace the eight-bit SAR ADC and achieve the same result of eight bits by restoring the results of the six-bit SAR ADC with a four-bit DAC. This structure can reduce the number of capacitors to one-fourth of the original structure, which can achieve an 80% power saving effect [119]. Multi-channel exchange arrangements with electrodes connected to ADCs via wires were often used in low-power ADCs, which were difficult to achieve a dynamic range with the medical reference value [134]. As an improvement, a multi-channel frequency modulated ADC (FM-ADC) was proposed [120]. Before being acquired by a single ADC, based on FM and frequency division multiplexing (FDM) techniques, the N switched channels are aggregated onto a single line by summing resistive current patterns. Through the multi-channel structure, the number of cables and ADCs was reduced, which in turn leads to a significant reduction in power consumption. A recent study [121] further improved the method of using V_cm_ for low-power processing, because the use of V_cm_ needs an additional low dropout regulator (LDO) which introduces additional power dissipation. Therefore, this research proposes a split capacitor-based switching scheme that requires less reference voltages than the traditional structure. This scheme reduces switching energy by 99.74% and saves the capacitor area by 48.44% compared to the traditional structure using V_cm_. Although this scheme significantly reduced the switching energy of the ADC, the researchers were not content with this. In another study [122], they proposed a switching scheme that comprehensively used a split capacitor array structure, MSB split method, and monotonic switching technique with bridge capacitors. As an improvement of the previous work, it saves 99.9% of the energy and reduces the number of capacitors by 96.9% compared with the traditional structure.

Adaptive algorithms can also effectively improve SAR ADC energy efficiency [123]. Unlike the general ADC, this device only outputs samples when the intensity of the signal exceeds a threshold. In this paper, a novel adaptive threshold algorithm is proposed, which can set the threshold level at an appropriate position within the dynamic range of the signal. It is called asynchronous because the sampling is non-uniform, and the sampling frequency depends on the number of quantization levels and changes with the amplitude of the input signal. The proposed SAR ADC compares the input signal with a threshold *L_n_* every *t_s_* seconds, where *Ln* belongs to the set *{L1, L2, ...* *Ln/V}*.

(7) $$\left(X_{\left(m-1\right)ts}-L_{n}\right)\left(X_{mts}-L_{n}\right)<0$$

(8) $$Q\left(s_{i}\right)=\left(m-1\right)t_{s}+t_{s}/2$$

(9) $$\lambda_{i}=L_{n\;}$$

When the Equation (7) is satisfied, the circuit detects the occurrence of crossover, which means that the crossover of the input signal and the threshold signal are the input in the period between $\left(m-1\right)t_{s}$ and $mt_{s}$. At this time, the quantized value $Q\left(s_{i}\right)$ (Equation (8)) of the time interval and the signal $\lambda_{i}$ (Equation (9)) that generates the crossover are recorded. Thus, this algorithm allows the ADC to achieve equivalent analog-to-digital conversion results while taking fewer samples under the same conditions, leading to lower power consumption and a smaller circuit area. Similar to this research, a dynamic circuit configuration method [124] was proposed for slowly changing single-channel signals such as the single-lead ECG and respiratory signals. In addition to the dynamic sampling frequency, adaptive resolution SAR ADCs can also be used [125]. It adopted the adaptive resolution ADC based on the fuzzy logic algorithm for the ADC in the wearable ECG monitoring system, which effectively reduces the circuit complexity and power consumption of the ADC. A similar design was employed in an EEG monitoring system [126].

The above strategies were targeted at the SAR ADC; a power minimization algorithm was proposed to lower the power consumption of the sigma-delta ADC [127]. It first confirms the oversampling rate that satisfies the dynamic range, and then finds the dc gain and stability time constant of the OPAMP by means of a behavioral simulator [135]. At the same time, the preset point of the minimum power can be deduced through the noise-and-settling constrained power minimization (NSCPM) algorithm. Further, simple sigma-delta ADCs with pipelined ADCs’ linear structure can perform complex work tasks. In the first conversion, the samples to be processed are input to the sigma-delta modulator. After the cycle, the MSB is extracted by the digital filter, while the residue enters the second conversion, and the LSB is obtained. Such a structure eliminates the need for the inter-stage amplification required by pipelined ADCs and achieves the desired resolution by digitally combining the MSB and LSB of the two conversion stages. Thus, two low-resolution, low-power sigma-delta modulators can also achieve high-resolution conversion using this architecture [128].

## 4. Conclusions and Outlook

In this review, we systematically summarize research on wearable sensors in the DAQ, which can be further divided into signal conditioning and analog-to-digital conversion. In the signal conditioning, amplifiers and filters are used to enlarge the target signal and minimize noise/artifacts, respectively. Recent amplifier designs pursue high positive feedback on differential mode signals while suppressing the common mode interference to achieve a higher CMRR, and utilize the characteristics of transistors operating in the subthreshold region to obtain high transconductance at low voltages. Three filters were used in different scenarios, such as to obtain a flat frequency spectrum through the Butterworth filter, to capture more details through the Chebyshev filter, and to reduce energy cost through the Elliptical filter. In addition, the adaptive filtering technology can effectively reduce the power consumption of the filtering circuit, and the sensor fusion algorithm centralizes the multi-sensor information to achieve the noise reduction effect. In the analog-to-digital conversion, reducing the capacitance mismatch and leakage current can effectively improve the linearity of the ADC, and various algorithms can reduce the number of capacitors or the electronic component discharging times to make ADCs more efficient.

It is worth mentioning that the signal conditioning unit and analog-to-digital conversion unit both play essential roles in the sensing system, but not each of them is indispensable. For example, the ADC can be replaced by specific algorithms. An ADC-free architecture was proposed in an ultra-low-power face recognition system [136]. In this structure, an analog convolution processor was used to perform the first layer quantization of the convolutional neural network (CNN), which can replace the traditional ADC to achieve the process. Compared with the traditional ADC structure, it can achieve similar results with a 15.7% reduction in power consumption. The similar design was demonstrated in other works [137,138]. However, such a structure has higher requirements on algorithms and circuits while only achieving limited improvement in energy efficiency. With the continuous improvement of the algorithm, this approach may become more efficient in the future.

There are research concerns to power consumption issues for every unit in the sensing system. Adoption of more advanced manufacturing processes [139,140,141,142], a simplified circuit [119,122], and other methods can significantly reduce the power of a certain unit of the sensing system. However, from the overall perspective of the sensing device, such improvement is relatively small. Taking the studies mentioned above as examples [143,144], they can bring microwatt levels of power reduction, while the power consumption of portable sensors is often at the milliwatt level. Alternatively, enhancing the power supply can solve the energy problem including using batteries with a high energy density [145] and fast charging capability [146,147], as well as applying self-powered designs that sensors obtain energy from the behavior of wearer [148,149], body temperature [150,151], body fluids [152], friction[153], raindrop [154], and sunlight [155]. In addition to using high-performance batteries, power management strategies provide support to improve the battery life of compact wearable sensors further [156]. Established power management strategies allow for waking up specific nodes of the sensor network [157] or turning on data transmission under ideal conditions [158]. They can also adaptively adjust the discharge power with the concentration of the object to be measured [159], and setting up non-volatile memory with sensor feedback from the object to be measured to extend the dormancy time [160]. In particular, self-powered batteries combined with power management strategies can maximize the reception of external energy and rationalize the output. For example, solar cells are partitioned according to the different body locations where the sensors are located to run all electricity at close to the maximum power point [161]. By analyzing the energy that self-powered loads receive from the outside world, the surplus is charged to the battery when there is enough energy, and the remaining battery energy is called to drive the sensor when there is not enough [162]. Meanwhile, the safety and portability of wearable sensors would not be compromised when the power consumption problem is solved.

Wearable sensors have broad development prospects and feasible research directions in terms of the DAQ. The large demand in health care, medical treatment, human-machine interface, and other fields will attract increasing attention in developing economical, efficient, and multifunctional wearable sensing devices.

## Figures and Tables

**Table 1:** Summary of signal amplification optimizations and their pros/cons in amplification capability for various applications.

Optimizations		Pros	Cons	Applications	Ref.
OTFT	Sub-thermionic	High gain; energy saving	Complicated operation	Healthcare; Human-machine interface	[36]
	Electron beam lithography	Low off current; quick switch	Low preparation efficiency	Healthcare; Emotion recognition	[37]
OECT	Cofacial vertical alignment	High gain; energy saving	Complicated operation; lot cost	Healthcare; Emotion recognition	[38,39]
Current Multiplexing Technology		Energy saving	Complicated operation	Healthcare	[40,41,42]
Post-mismatch compensation technology		Transistor mismatch reduction	Complicated operation	Healthcare	[43]
Common-mode feed-forward technique		CMRR improving	Complex structure	Healthcare; Emotion recognition	[44]
Time division multiplexing		Impedance mismatch reduction	Accurate clock source needed	Healthcare; Environmental analysis	[45,46,47]
Copper stabilization technique		Low frequency noise reduction	Noise frequency limitation	Human-machine interface	[48,49]
Fully differential difference amplifier		Transistor mismatch reduction	Area needed	Healthcare	[50]

**Table 2:** Summary of noise filtering optimizations and their pros/cons in filtering capability for various applications.

Optimizations	Pros	Cons	Applications	Ref.
Butterworth filter	Passband flat	Long stopband	Healthcare Motion capture	[74,75,76,77]
Chebyshev filter	Short stopband	Passband ripple	Healthcare Emotion recognition	[78,79,80,81]
Elliptic filter	Short stopband	Passband ripple	Healthcare Human-machine interface	[82,83,84]
Adaptive filter structure	Large filter range Targeted noise filtering	Complex algorithms	Healthcare	[85]
Particle filter	Precise filtering	Complex algorithms	Location tracking	[86,87]
Kalman filter	Multi-sensor fusion	Complex algorithms	Healthcare Emotion recognition Human-machine interface	[88,89]

**Table 3:** The improvement of the SNR and the PSD by the three types of filters.

Filter	SNR before Filtering (dB)	SNR after Filtering (dB)	PSD before Filtering (dB/Hz)	PSD after Filtering (dB/Hz)
Butterworth filter	−5.5313	11.8139	−54.8834	−68.5366
Chebyshev I filter	−5.5313	9.0951	−54.8834	−70.3884
Elliptic filter	−5.5313	12.2930	−54.8834	−70.9543

**Table 4:** Summary of analog to digital conversion optimizations and their pros/cons in converting capability for various applications.

Optimizations	Pros	Cons	Applications	Ref.
mini-c-DAC assist	leakage current reduction	Complex structure	Human-machine interface	[108]
Tracking algorithm and NSU dither jitter injection method	Noise and capacitance mismatch	Complex algorithms and structure	Healthcare Human-machine interface	[109]
Capacitor reorganization method	Nonlinear reduction	Energy consumption increased	Healthcare	[110]
Minimum error algorithm	Nonlinear reduction	Complex algorithms	Healthcare	[111]
Sigma-delta modulation combining	Nonlinear reduction; Resolution improvement	Complex algorithms and structure	Healthcare Emotion recognition Human-machine interface	[112,113,114,115]
Compressive sensing	Sampling frequency reduction	Slow sampling	Healthcare Emotion recognition	[116]
Bypass-switched structure	MSB conversions reduction	Slow sampling	Healthcare	[117]
LSB priority algorithms	Low precision ADC	Complex algorithms	Emotion recognition	[118,119]
Multi-channel exchange arrangement	Reduce power consumption of cables	Complex equipment	Healthcare	[120]
Dynamic Capacitive Switching	Intermittent work	Complex structure; Low precision	Healthcare	[121,122]
Adaptive threshold algorithm	Intermittent work	Complex algorithms; Slow sampling	Healthcare Emotion recognition Motion capture	[123,124]
Adaptive resolution algorithm	Low resolution conversion noise	Complex algorithms Low noise signals only	Healthcare	[125,126]
Power minimization algorithm	Low resolution for high accuracy conversion	Complex algorithms Slow sampling Complex structure	Healthcare	[127,128]

## Data Availability

Not applicable.

## Abbreviations

DAQ	Data acquisition
DT	Data transmission
ADC	Analog-to-digital convertor
CMRR	Common-mode rejection ratio
SAR	Successive approximation register
FET	Field effect transistor
TFT	Thin film transistor
OTFT	Organic thin film transistors
OECT	Organic electrochemical transistors
EEG	Electroencephalogram
ECG	Electrocardiogram
SAM	Self-assembled monolayer
PaC	Parylene C
NMOS	Negative metal oxide semiconductor
PMOS	Positive metal oxide semiconductor
PMC	Post-mismatch compensation
CMFF	Common-mode feed-forward
TDM	Time division multiplexing
AFE ASCI	Analog front-end application specific integrated circuit
PPG	Photoplethysmography
FDDA	Fully differential difference amplifier
OPAMP	Operational amplifier
OTA	Operational transconductance amplifier
DDA	Differential difference amplifier
CMFB	Common mode feedback
PSoC	Programmable embedded system-on-chip
VGA	Variable gain amplifier
SNR	Signal-to-noise ratio
CF	Complementary filtering
DAC	Digital-to-analog converter
SFDR	Spurious-free dynamic range
SNDR	Signal-to-noise distortion ratio
LSB	Least significant bit
CS	Compressive sensing
MSB	Most significant bit
